# Wettability of Asphalt Concrete with Natural and Recycled Aggregates from Sanitary Ceramics

**DOI:** 10.3390/ma13173799

**Published:** 2020-08-28

**Authors:** Wojciech Andrzejuk, Andrzej Szewczak, Stanisław Fic, Grzegorz Łagód

**Affiliations:** 1 Faculty of Technical Sciences, Pope John Paul II State School of Higher Education in Biała Podlaska, Sidorska 95/97, 21-500 Biała Podlaska, Poland; 2 Faculty of Civil Engineering and Architecture, Lublin University of Technology, Nadbystrzycka 40, 20-618 Lublin, Poland; a.szewczak@pollub.pl (A.S.); s.fic@pollub.pl (S.F.); 3 Faculty of Environmental Engineering, Lublin University of Technology, Nadbystrzycka 40B, 20-618 Lublin, Poland; 4 Faculty of Civil Engineering, Czech Technical University in Prague, Thákurova 7, 166 29 Prague 6, Czech Republic

**Keywords:** aggregate from sanitary ceramic wastes, mineral–asphalt mixtures, surface free energy, wettability, porosity

## Abstract

In line with the current trend of seeking alternative methods for modification of the existing building composites, such as mineral–asphalt mixtures (MAMs), the materials from concrete and ceramics recycling are being used in increasingly wider applications. When added to MAMs as an aggregate, ceramic building material, which has different properties than the raw material (clay), may significantly influence the aggregate properties, including the wettability, porosity, asphalt adhesion, and consequently the mixture durability. The material’s microstructure was found using SEM. The wetting properties of mineral–asphalt mixtures were determined by measuring the contact angles (CA) of their surfaces, using water as the measuring liquid. The total surface free energy (SFE) values were determined using the Neumann method. When analyzing the research results, it can be noticed that the chemical composition of the ceramic aggregate has a significant influence on the adhesion of asphalt to its surface due to the chemical affinity. Waste ceramic aggregate, despite its acidic pH value being connected with its elevated silica content, exhibits good adhesive properties.

## 1. Introduction

Civil engineering and water engineering are branches of the global economy, which contribute to the emission of significant amounts of CO_2_ and the consumption of energy in the form of building material production and building construction processes [1,2,3,4]. These branches also dynamically develop new technologies, aiming to prevent the unfavorable impacts of the above processes on the natural environment. The main criteria, which have to be met by buildings due to sustainable development policies, include durability, reliability, and reduction of the energy consumption during the material manufacturing stage, as well as during building construction and operation [5,6,7,8]. The application of recycled products and additives is currently one of the most popular methods of modifying building materials. The waste materials from industry, households, agriculture, and other sectors of the economy are subjected to processing, e.g., grinding, crushing, melting, separation of chemical compounds and substances [9]. Depending on the properties that characterize them, the obtained products can be used as additives in building materials, mainly in composites. The waste aggregates from agricultural production are a good example, which may be used as fillers in cement–lime composites, improving the thermal insulation of building barriers [10]. Waste processing is highly important [11]; therefore, the ongoing research aimed at optimizing the recycling process and improving its efficiency is mainly focused on the possibility of reusing the greatest possible amount of wastes, the production, transport, storage, and disposal of which are significant issues [12,13,14,15,16]. The growing amount of waste, combined with the lack of the possibility to quickly replenish the sources of natural raw materials (including aggregates), necessitates the search for the methods that can be used for their recovery and reuse. Nevertheless, appropriate modification of the properties characterizing construction composites enables the efficient application of recycled materials [17,18].

The considered materials include fly ash materials from the combustion of coal, biomass, and other fuels in power plants [19,20]; as well as the sludge materials from wastewater processing, which are used as concrete additives (normal, asphalt, high-grade) and in mineral–asphalt mixtures (MAMs) [21,22,23,24,25]. The main aim of utilizing these additives is to reduce the amount of voids in the material, thus decreasing the porosity and absorptivity, as well as improving the durability. As a consequence, the potential exploitation time for buildings and other infrastructure objects constructed using these materials is extended. The oil substances originating from the crude oil refining process and the food industry are other additives that can be used to modify the structures of building materials (including asphalt) and in temperature regulation (thereby reducing the energy required for the preparation and processing of mineral–asphalt mixtures) [26,27,28]. The application of shredded rubber (e.g., from spent tires) as an asphalt and mineral–asphalt mixture (MAM) filler that influences the frost resistance, rigidity, durability, and resistance to road surface damage is also common [29].

For composites, which also include the mineral–asphalt mixtures, one of the current main research directions is the search for alternative aggregates that conform to the sustainable development principles [16,30]. In traditional mineral–asphalt mixtures, the most commonly employed aggregates are crushed aggregates (granite, dolomite, basalt, granodiorite), pebble aggregates, and crushed asphalt. The intended applications of the mixtures are relevant (base layer, wearing course). These aggregates are characterized mainly by their large specific surface area and capacity to absorb asphalt on their surfaces, which stems from their surface porosity. Maintaining continuous granulation is also an important aspect. Nevertheless, obtaining these types of aggregates necessitates interference into the natural resources; therefore, substituting the natural aggregates with aggregates from other sources is important both financially and from the point of view of implementing sustainable development principles into a circular economy [30]. Therefore, the possibility of obtaining such aggregates from recycled wastes is being tested. These aggregates can be characterized by their diverse chemical and phase compositions, as well as their origins, including [31,32,33,34,35]:

- Aggregates obtained through secondary crushing of ceramic and glass waste materials;
- Aggregates obtained from crushing or grinding of concrete, debris, and appropriate types of ground materials;
- Aggregates obtained from crushing asphalt surfaces and other road layers;
- Aggregates obtained from the grinding and processing of other materials, such as plastic, rubber, and composites.

These types of aggregates can significantly influence the properties of concretes and mineral–asphalt mixtures by minimizing the consumption of traditional aggregates, improving the strength and durability as a result of the occurrence of variable conditions (i.e., humidity and temperature, water absorption, changes to the mechanical strength, including resistance to dynamic stress), and increasing the adhesion of the asphalt binder or cement paste to the aggregate [31,32,33,34,35]. Some materials, including ceramics, glass, and debris, cannot be utilized in primary production following their use; in such cases, their reuse is not always possible. When opting for the application of waste aggregates in mineral–asphalt mixtures, asphalt concretes, or polymer concretes, the specific parameters that are required of the mixture need to be considered. This necessitates the adoption of certain assumptions when designing the composite based on the known or predicted properties of the waste aggregates. For MAMs, the important aspects include the adhesion of the asphalt to the aggregate, void content, resistance to plastic deformation, water absorption, wettability, workability, mixture preparation temperature, and content of external materials [36,37,38]. Ceramic waste materials are one type of waste material used for these purposes. Heating clay (e.g., into bricks) causes irreversible changes in its structure and transforms it into products with diverse physical and mechanical properties. These changes prevent the restoration of the initial parameters of clay following the recycling process [31,32,33,34,35].

The adhesion of asphalt to the aggregate depends on numerous factors. The basic parameters determining the suitability of an aggregate for a mineral–asphalt mixture are its wettability and roughness [39,40]. If a surface is uneven this improves the mechanical adhesion between the binder and the surfaces of the aggregate grains. The contact angle (CA) and surface free energy (SFE) are the parameters that describe these phenomena in a detailed way [39,40,41,42,43]. There are several methods for determining these parameters, e.g., the Owens–Wendt, Neumann, Fowkes, Wu, Zisman, and van Oss–Chaudhury–Good methods [39]. Each of these methods involves the determination of the contact angle of the considered surface by liquids with known dispersion–polar surface tension properties. Depending on the formulas assumed in a given method, one, two, or three measurement liquids may be involved, which can include distilled water, glycerin, diiodomethane, glycol, and formamide.

Roughness is a parameter that directly defines the mechanical adhesion to the aggregate surface. The most popular method for determining the roughness parameters involves tests conducted using a profilometer and a microscope. By knowing the general profile image of an aggregate’s surface, it is possible to determine its smoothness, the amount of depressions, the mean height of the bumps, and the surface porosity [39,40].

An important parameter that also largely influences the adhesion of the asphalt to the aggregate is the aggregate’s viscosity at the moment of mineral–asphalt mixture preparation—depending on the method, this can be when the aggregate is cold, warm, or hot. Viscosity, which is a measure defining the cohesion of hydrocarbon particles in a bituminous mass, largely depends on the temperature and presence of particular function groups [41]. It is possible to regulate the asphalt’s viscosity using additives, including a large number of polymer additives, which may alter the output parameters of the asphalt in a significant way. Regarding the preparation of mineral–asphalt mixtures, the utilized modifications should be aimed at improving the viscosity while simultaneously maintaining the possibility of mixing asphalt with aggregate, as well as improving the bonding time [42,43].

In civil engineering, ceramics are commonly employed in concretes and mortars [44,45,46,47]; however, there is a lack of publications that precisely describe the parameters and conditions needed to apply these aggregates in the mixtures used in the road construction industry. A significant problem may be to determine the optimal amount of added recycled aggregate so that it does not significantly deteriorate the properties of the mixture [18,48].

The solution analyzed in this paper corresponds to the application of a crushed ceramic as a composite filler in a mineral–asphalt mixture. An aggregate made from waste enamel ceramic is added to a mineral–asphalt mixture as a partial substitute for traditional dolomite aggregate.

Mixtures of composites are designed for use in road engineering. On the basis of the previous research [48], the content of the waste ceramic aggregate in the prepared mineral–asphalt mixtures is approximately 30%. A mixture entirely comprising traditional dolomite aggregate is used as a reference.

## 2. Materials and Methods

### 2.1. Material Properties

Two mineral–asphalt mixtures containing different types of aggregates were used for the study: one with dolomite as the aggregate traditionally, which is used in bituminous surfaces; and one with waste ceramic aggregate as a partial substitute for the aggregates traditionally used in mineral–bitumen mixtures. Sanitary ceramic waste with dimensions of about 20 cm × 40 cm was transported to the laboratory and initially crushed in a ball crusher to a size range of 2–4 cm. This material was then subjected to crushing in a jaw crusher.

An analysis of basic technical parameters of examined aggregates is presented in Table 1 [47].

On the basis of the earlier studies [48], it was assumed that 20–30% of the waste ceramic aggregate addition can be used as a substitute for the aggregates traditionally used in mineral–asphalt mixtures for road surface construction.

Road bitumen 50/70 was used as a binder in the mineral–asphalt mixtures. The binder parameters are presented in Table 2.

### 2.2. Methods

The test program outlined the preparation of the mixtures intended for the wearing course (WC). Two series of mixtures were proposed. The first series mixture (WC-1) was made using dolomite and ceramic aggregates, while the second series mixture (WC-2) contained only dolomite aggregate. It was assumed that the laboratory recipe for WC would be developed for the asphalt concrete intended for the wearing course, with a grain size range of 0–11.2 mm, for use with surfaces with category TL1 ÷ 2 traffic loads (Table 3). The formulas were developed based on the regulations in [36,37]. The mixtures were made at three different temperatures: 140, 150, and 160 °C. The final mixture compositions are presented in Table 4.

The densities of the mineral asphalt mixtures were determined in accordance with the regulations in [38]. It was assumed that the mass of the analyzed specimen expressed in grams should be at least 50 times larger than the thickest grains of the aggregate found in the mineral–asphalt mixture. The specimens were crushed into respective grains or clusters so that the diameters of the clusters did not exceed 6 mm. In the case of the compacted specimen, it was placed in a dryer with an operating temperature of 110 °C and heated until crushing was possible. The next test involved the determination of the bulk density of the mineral–asphalt mixture. The test was conducted with the use of Marshall specimens. Each specimen was weighed and immersed in a water bath for 40 min.

The contact angle (CA) characterizing the liquid drop was measured on a research setup comprising a goniometer and a camera used to capture images of single drops put onto the surface of each sample. The analysis of the contact angle was conducted with distilled water. The wetting angles, θw, which corresponded to the surface coatings, were measured with a liquid characterized by known total surface free energy (SFE) values (γw). The constant volumes of liquid drops measuring approximately 2 mm^3^ were applied onto the sample surfaces via a micropipette. Three drops were applied onto each mineral–asphalt sample. Since the aggregate was covered in asphalt, adsorption was not taken into consideration. The measurements were conducted at a temperature approximating 22.5 °C at the moment each drop was applied.

A Neumann model, which constitutes one of the most common methods for calculating SFE, was used in order to determine this parameter. The employed equation was as follows [50]:

(1) $$\cos\theta_{w}=[e^{-0.000125{\left(\gamma_{s}-\gamma_{w}\right)}^{2}}\cdot 2\sqrt{\frac{\gamma_{s}}{\gamma_{w}}}-1]$$

where $\gamma_{s}$ is the total SFE; $\gamma_{w}$ is the SFE of water, which equals 72.8 (mJ·mm^−2^); and $\theta_{w}$ is the water contact angle.

One of the tests involved the dynamic viscosity of the asphalt. The dynamic viscosity is a coefficient of internal friction, which is created when two parallel layers of the tested material (asphalt) move against each other at a certain temperature. The unit for dynamic viscosity is Pa∙s. Dynamic viscosity is one of the most important parameters for the assessment of asphalt behavior for long-term road surface loads, as well as one of the most important technological features on which the conditions of production, mixing, and transport of mineral–asphalt mixtures depend [51]. The dynamic viscosity was measured in accordance with EN 13302 [52]. The test was carried out using a Brookfield AMETEK Thermosel (Middleboro, MA, USA) system consisting of a Brookfield AMETEK viscometer (DV3T) with related accessories to accurately measure the viscosity levels of liquids at elevated temperatures.

A scanning electron microscope (Quanta FEG 250 microscope by FEI, Hillsboro, OR, USA) equipped with a system used for the chemical composition analysis based on energy-dispersive X-ray spectroscopy (EDS), manufactured by EDAX (Mahwah, NJ, USA), was employed to determine the morphology and structure of aggregates.

The Hommel-Etamic T8000 RC120-400 (Jena, Germany) modular device was used to measure the contour, roughness, and 3D topography. The roughness and contour features were assessed using a uniform user interface, allowing the calculation of all normalized parameters of the roughness and waviness profiles and the assessment of geometric features such as distances, angles, and radii. For topography measurements, the set was additionally equipped with a CNC (Computerized Numerical Control) table that allowed control of the movement of the measured part in the Y direction.

## 3. Results

**Figure 1:**
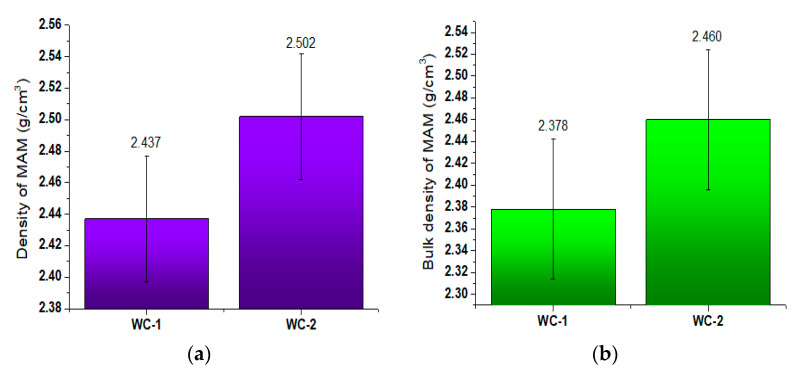
Density and bulk density values for mineral–asphalt mixtures. (**a**) Density; (**b**) bulk density.

The results for the mineral–asphalt mixture density and bulk density are presented in Figure 1.

**Figure 2:**
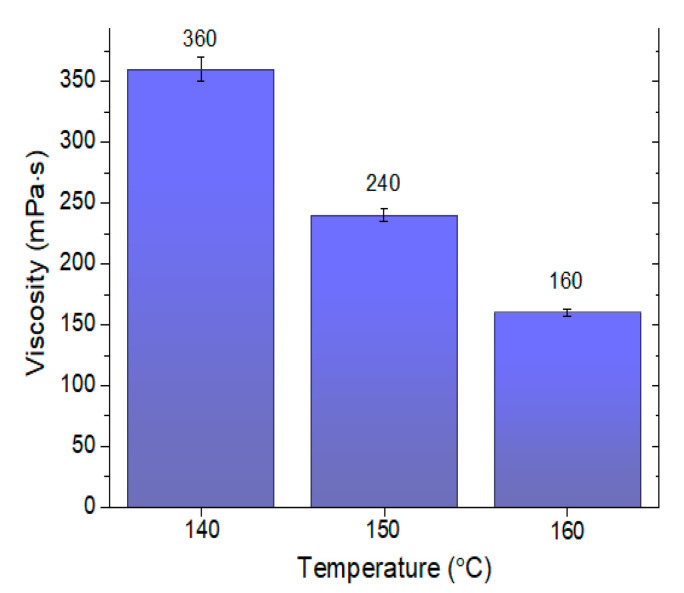
Viscosity of 50/70 road asphalt.

The results of the 50/70 road asphalt viscosity measurements at three different temperatures are shown in Figure 2.

**Figure 3:**
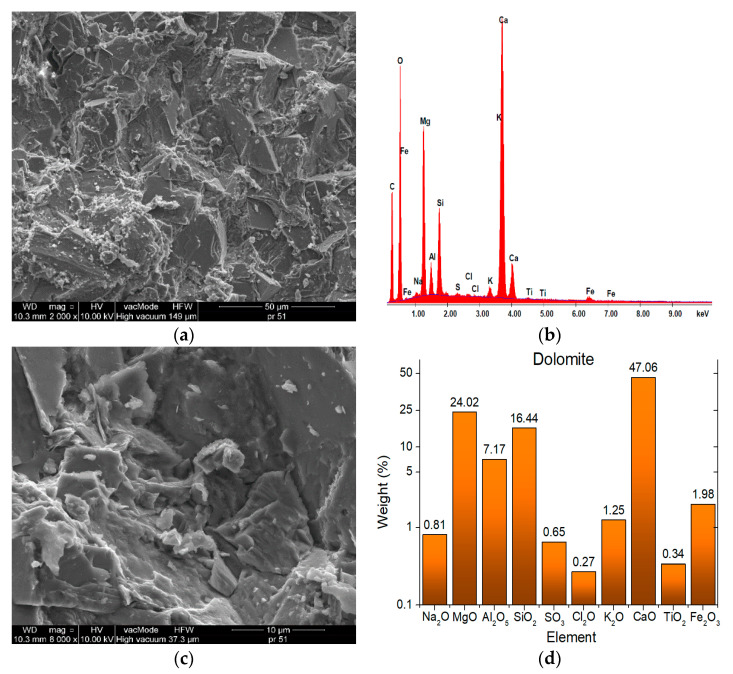
Dolomite SEM microstructure (2000× and 8000×) and elemental analysis results in the EDS micro area. (**a**) SEM microstructure (2000×); (**b**) composition; (**c**) SEM microstructure (8000×); (**d**) percentage composition.

**Figure 4:**
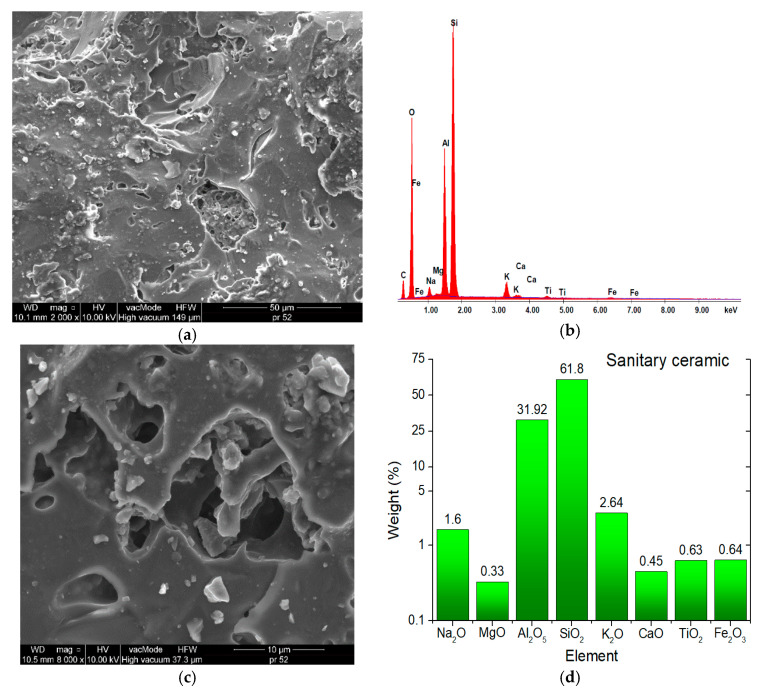
Sanitary ceramics SEM microstructure (2000× and 8000×) and elemental analysis results in the EDS micro area. (**a**) SEM microstructure (2000×); (**b**) composition; (**c**) SEM microstructure (8000×); (**d**) percentage composition.

**Figure 5:**
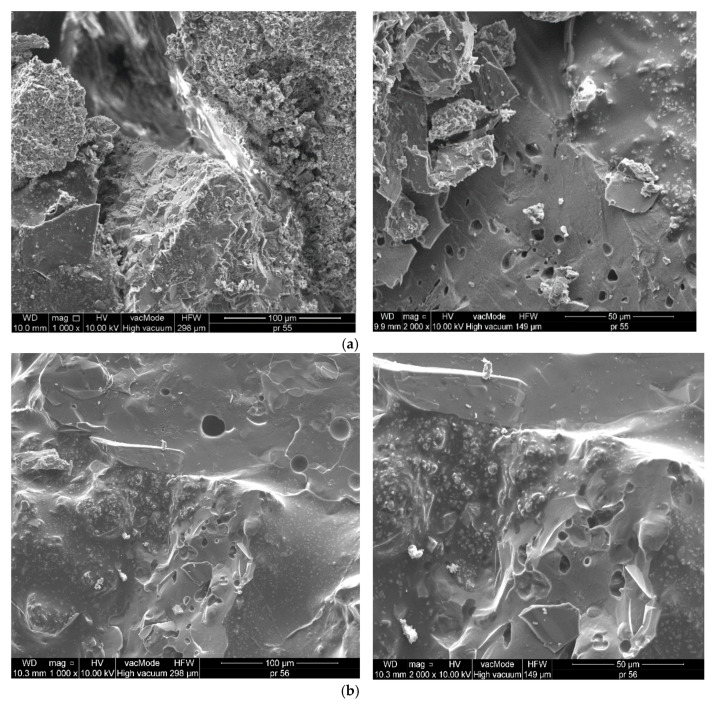
SEM microstructures of mineral–asphalt mixtures (1000× and 2000×): (**a**) WC-1; (**b**) WC-2.

Figure 3 and Figure 4 present the SEM images showing the microstructures of aggregates, while Figure 5 demonstrates the SEM images showing the microstructures of mineral–asphalt mixtures. The chemical compositions of the aggregates are presented based on energy dispersive spectrometry (EDS).

**Figure 6:**
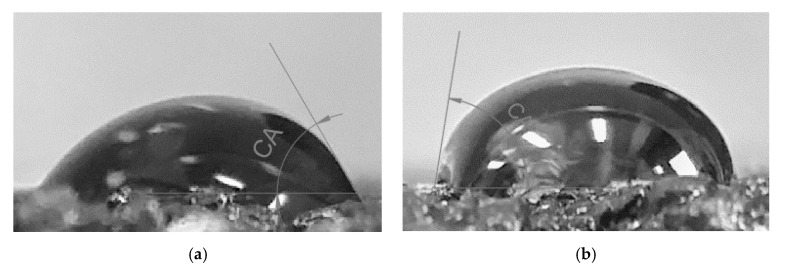
Contact angle of a water drop in mineral–asphalt mixtures: (**a**) WC-1; (**b**) WC-2.

Figure 6 presents the exemplary photos of CA water measurements of WC-1 and WC-2 mineral–asphalt mixtures. The SFE values for all analyzed bituminous mixes were calculated by means of Neumann’s method on the basis of the CA measurements.

Table 5 and Table 6 present the CA values for water measured on each mineral–asphalt mixture (determined in ten points) and the SFE values calculated from the results.

**Figure 7:**
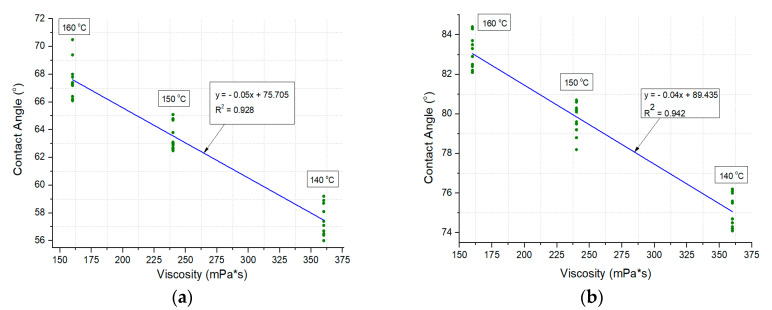
The correlations between the viscosity and contact angle values in mineral–asphalt mixtures: (**a**) WC-1; (**b**) WC-2.

**Figure 8:**
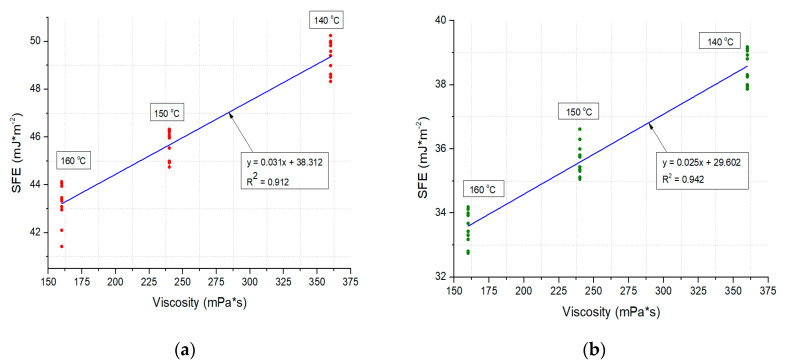
Correlations between viscosity and surface free energy values in mineral–asphalt mixtures: (**a**) WC-1; (**b**) WC-2.

Linear correlations between the asphalt viscosity and contact angle, as well as between the viscosity and surface free energy, were observed. The correlations are presented in Figure 7 and Figure 8.

**Figure 9:**
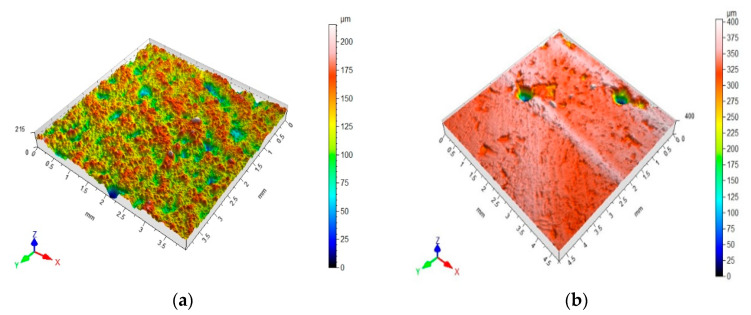
Microroughness values and representative profilograms showing the surfaces of mineral–asphalt mixtures (MAMs): (**a**) WC-1 (160 °C); (**b**) WC-2 (160 °C).

Microroughness values and the representative profilograms showing the surfaces of the mineral–asphalt mixtures are presented in Figure 9. The measurements were performed in line with the EUR15178N standard.

**Figure 10:**
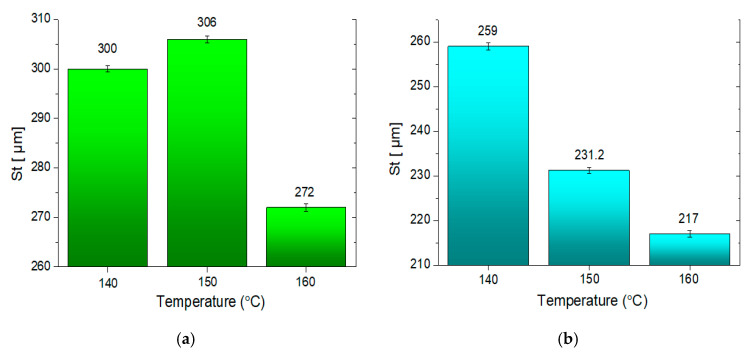
The total height (St = Sp + Sv) values of the mineral–asphalt mixtures: (**a**) WC-1; (**b**) WC-2.

The roughness characteristics obtained for the tested mineral–asphalt mixtures are presented in Table 7, as well as in Figure 9 and Figure 10.

## 4. Discussion

In order to explain the phenomena occurring in the structures of mineral–asphalt mixtures, the physicochemical properties of the utilized aggregates and asphalt have to be considered. The analysis of SEM images provides the information that can explain the manner of the adhesion of the asphalt binder to the aggregate surface.

Figure 3 and Figure 4 present the photographs showing the surfaces of both employed aggregate mixtures, which differ in texture and surface structure. As a result of crushing, the dolomite pebbles are characterized by larger surface indentations and bumps in relation to the porous and flat surfaces of ceramic grains. The model of the mechanical interlocking connection presented in [53,54] can be used for the description of the adhesion in the contact zone, involving an aggregate with a diversified surface and a binder. The basic assumption in this model involves the mechanical adhesion to irregular rough surfaces. In the considered case, mechanical interlocking occurs in the grain–asphalt binder contact zone in both employed aggregates. Moreover, the authors in [55,56] observed that the bonding between the asphalt and aggregate results from three basic factors, one of which, pertaining to the surface texture and aggregate porosity, is the effect of physical phenomena. These aspects will be described further on. The other two factors are the aggregate’s mineralogy and adsorption of appropriate ions on its surface, which affect the chemical phenomena during the bonding of asphalt with the aggregate. According to [42], the developed specific surface, while maintaining the constant volume condition, has the greatest influence on the coverage of the aggregate surface by the asphalt. Therefore, the asphalt-covered ceramic aggregate has higher surface energy than the dolomite aggregate.

Analysis of the chemical compositions of aggregates provides data on the external surface structures and their chemical affinity towards asphalt. Due to the acidic pH of the asphalt binder resulting from the large number of carboxyl groups, which are components of macromolecular hydrocarbon chains, a greater chemical affinity is exhibited towards alkaline aggregates (dolomite has high contents of calcium, magnesium, iron, and aluminum) [18,48,57]. Therefore, the application of the dolomite aggregate is more favorable in this case, because more durable compounds can be obtained at the phase interface (interface between liquid asphalt and solid carbonate aggregate) [58]. These reactions result in the better adhesion of asphalt to the aggregate. However, the possibility of using a waste ceramic aggregate in MAMs should not be ruled out. The chemical composition and surface structure are not the only criteria for determining its usefulness. The obtained results confirm certain assumptions found in the literature. Apart from the roughness and chemical composition, the important parameters that affect the adhesion quality include the wettability and rheological properties of asphalt [59]. The conducted studies indicated that the presence of water in the asphalt mixture facilitates its migration from the binder to the surface pores. In this way, water with fine asphalt particles contributes to better fixation of the pores of the ceramic grains to the indentations on the dolomite surfaces. The bonding process between asphalt and aggregate largely depends on the rheology of the asphalt mixture (see Figure 2). The research of [55,56] should also be discussed. Asphalt adhesion in the presence of water is reduced along with an increase in pH. This is because of the separation of asphalt from the aggregate in the course of its ageing during operation. The hydrogen ions from water can be transferred onto the aggregate surface, penetrating the thin layer of asphalt on its surface [56]. This results in an increase of pH, whereas the asphalt is displaced and removed. This process can be mitigated by the presence of powder additives, including dolomite or gravel meal [60]. In this case, an increase in pH is stabilized so that it stops after some time. Therefore, the addition of ceramic aggregate, characterized by pH values ranging from 3 to 5 (acidic), is justified. In this case, the affinity of hydrogen ions from water and their tendency towards adsorption on the aggregate structure will be lower.

The data presented in Figure 3 indicate that despite the alkaline pH of the aggregate, dolomite contains a relatively high amount of SiO_2_ (16.4% at 61.8% of ceramic aggregate). This may mean that stronger chemical interactions will occur in the mixture of these aggregates with asphalt and that the relevance of the polar constituent of the SFE will increase.

Figure 5 shows the air bubbles in the asphalt mixture, which were created in the course of mechanical mixing of the components. Air bubbles, as a result of mixing, tend to stick to the grain surfaces, especially in the surface indentations [40,61].

In other works [54,61], it was observed that non-rinsed aggregate grains absorb the silty particles occurring in the course of mechanical crushing. The presence of silty particles, which pollute the grain surfaces, hinders wettability; the contact angle in this scenario was approximately 45% greater in relation to those observed on the surfaces of the rinsed grains. The presence of air bubbles and pollutants lowers the adhesion in the contact zone, in line with the weak boundary layer model and Bikerman’s classification. Bikerman’s model, although criticized, accounts for the poor adhesion in the indicated areas as weak interfacial bonding [62].

Kinloch [63] indicated that the presence of air bubbles, microscratches, and sharp surface unevenness are the defects that hinder and weaken the mutual bonds, including the chemical bonds. This phenomenon should be accounted for during the analysis of bonding with dolomite pebbles, due to the presence of numerous surface indentations.

In the WC-1 mixture, 52% more limestone dust was used in order to raise the alkalinity of the mixture of dolomite and ceramic aggregates. Despite the high content of silicon having an influence on the acidic pH of the ceramic aggregate, it should be remembered that silicon—being the fourth main group element—may transfer some of the free electrons to the layer of delocalized electrons, which belong to aromatic hydrocarbons [64]. The efficiency of the coverage of the aggregate with asphalt binder can also be improved by utilizing adhesive or polymer agents, which is currently a common practice during MAM preparation [43,65].

The ceramic aggregates are characterized by having greater strength and durability compared to the dolomite aggregate. Depending on the origin, the ceramic aggregates have 3- to 10-fold greater compressive strength (Table 1). Moreover, as indicated by the analysis of SEM images, the aggregate grains are irregular, with uneven surfaces, bumps, and depressions, which positively affects the asphalt layer adhesion (Figure 4). The MAM preparation temperature is also relevant in this case. With increasing temperature, the asphalt viscosity is reduced (at a temperature of 160 °C it is 2.25-fold lower than at 140 °C) (Figure 2). This facilitates the distribution of the binder over the aggregate, filling the irregularities, depressions, and concavities. The lower bulk density resulting from the presence of voids, which can be filled in by asphalt of lower viscosity, is also important in this case (Figure 1). A thorough covering of the ceramic aggregate with asphalt is also favorable in terms of decreasing the absorptivity of the aggregate. As can be seen in the presented SEM images (Figure 3 and Figure 4), numerous pores and capillaries that facilitate water infiltration are visible on the ceramic aggregate surface. In the case of the dolomite aggregate, the number of pores is lower (Table 1), whereas the absorptivity of this aggregate results from the calcium carbonate and magnesium carbonate structures, which are the main components of dolomite.

On the basis of the conducted literature analysis, it can be stated that in the mixtures with aggregate filler, one can observe the wall effect [66], loosening effect [67,68], and wedging effect taking place, which are caused by the diversified diameters of aggregate grains in the mixture [68,69]. These phenomena can be caused by the presence of a filler and probably had an influence on the compaction of components in the considered mixtures, as indicated by the density measurements (Figure 1)

The SFE analysis enabled statements to be formed pertaining to the wettability of the WC-1 and WC-2 mixtures. For both mixtures, a constant increase in CA along with temperature was noted in the experiment, translating into lower SFE values, which is an indicator of wettability in the case of the mineral–asphalt mixtures (Table 5 and Table 6; Figure 7 and Figure 8). The unfavorable phenomenon involving leaching of asphalt on the aggregate surface by water, which may be aggravated by the presence of compounds of sulfur, nitrogen, or heavy metals, is also worth mentioning.

In the WC-1 mixture, the 18% increase in the CA reduced the SFE by 14%. This is related to the coverage process of the aggregate with asphalt (it should be mentioned that the amount of ceramic aggregate in relation to the dolomite aggregate in the WC-1 mixture amounts to 56.1%). The SEM images show a greater amount of surface pores in this mixtures, which contribute to the better adhesion of water to the mixture’s surface (some of the water directly infiltrates into pores, despite their partial filling with asphalt, which is more pronounced the lower the asphalt viscosity). Therefore, the contact angle is greatest at a temperature of 160 °C.

Similar correlations were observed in the case of the WC-2 mixture. In comparison with the WC-1 mixture, the value of the mean contact angle is 30% greater at a temperature of 140 °C and 23% greater at 160 °C. These results translate into a 28% reduction in the SFE, regardless of the experimental temperature, which stems from the better adhesion of asphalt to the WC-2 mixture. It should be remembered that the SFE is also a parameter describing the adhesion of the investigated measurement liquid on the surface of the target substrate [70,71]. Therefore, if an appropriate technological regime is maintained in the MAM production, accounting for the higher temperature of the asphalt preparation and the application of adhesive agents and limestone dust, utilization of the waste ceramic aggregate may contribute to the production of more durable mixtures [42,43].

The last factor that should be taken into account, which is either directly or indirectly connected with the abovementioned observations, is the surface roughness of the obtained mineral–asphalt mixtures. The results presented in Figure 9 and Figure 10 and Table 7 provide the answers to the issues connected to the coverage of aggregate surfaces by the asphalt. The lower viscosity of the asphalt (at the highest temperature of 160 °C) facilitates its infiltration into the pores, depressions, and bumps in the aggregate’s structure. The differences in the Sp and Sv values stem from the more diversified surface porosity and roughness of the ceramic aggregate. While analyzing the surface parameters, it should be observed that the depressions on the aggregate’s surface are filled more thoroughly. This is evident in the results pertaining to St, which is an amplitude expressing the coverage of the aggregate surface by asphalt. The differences between the WC-1 and WC-2 mixtures stem from the presence of the ceramic aggregate in the former. Increased temperature during asphalt preparation is beneficial to improving the adhesion resulting from the physical phenomena (simultaneously lowering the water absorption on the surface of the MAM), whereas the temperature of the asphalt is important for increasing the adhesion connected with the chemical phenomena and adsorption of ions on the aggregate structure.

In summary, the application of the ceramic aggregate as a substitute for the dolomite aggregate is justified for two reasons. The first is the possibility of improving the mechanical adhesion of the asphalt to the aggregate through the aggregate’s increased roughness and through the addition of asphalt heated to a higher temperature (in the case of the conducted investigations, this temperature was 160 °C). These factors improve the mixture’s resistance to the unfavorable effects of water resulting from the separation of asphalt from the aggregate. The second method involves limiting the pH increase on the aggregate surface through the application of ceramic waste, which is characterized by acidic pH values. This enables the preparation of asphalt at lower temperatures and improves the chemical adhesion. The possibility of increased water adsorption on the surface of the MAM is a side effect; however, the presence of an aggregate with low pH prevents the infiltration of hydrogen ions from water to the aggregate surface. Thus, this phenomenon is eliminated.

## 5. Conclusions

On the basis of the obtained research results, the following conclusions were drawn:

1. The chemical composition of the ceramic aggregate has a significant influence on the adhesion of asphalt to aggregate’s surface due to the chemical affinity; however, this parameter is not decisive. Waste ceramic aggregate, despite its acidic pH connected with the elevated silica content, exhibits good adhesive properties, which are connected with the electron structure of asphalt; it is also possible to raise the alkalinity and adhesion of this aggregate by increasing the addition of limestone dust or adhesive agents;
2. Waste aggregate, owing to its higher compressive strength (about 3–10 times higher than dolomite aggregate), increases the durability of the wearing course produced using the MAM prepared with the WC-1 recipe;
3. It is recommended to prepare the MAM with waste ceramic aggregate addition at elevated asphalt heating temperatures (150–160 °C), which reduces the asphalt viscosity, enabling more thorough coverage of the aggregate grains;
4. Due to the higher porosity of ceramic aggregate grains, the mixture with the addition of waste aggregate is characterized by higher wettability, which may lead to the leaching of the asphalt binder on the aggregate surface. In order to unequivocally evaluate the long-term effects of this phenomenon on the aggregate surface, further studies should be conducted;
5. The surface parameters of the investigated samples describe the results of the physical phenomena involving the filling of an uneven aggregate surface by asphalt. The parameters also explain the influence of aggregate roughness on the adhesion of the asphalt;
6. The chemical phenomena and the creation of bonds between the aggregates and the asphalt result from the chemical compositions of aggregates, the pH values of the surfaces of MAM components, and the adsorption of particular ions on the aggregate surfaces.

## Figures and Tables

**Table 1:** Technical parameters of aggregates used in the analysis based on [47].

Parameter	Dolomite	Sanitary Ceramics
Specific density (g/cm^3^)	2.4–2.8	2.64
Bulk density (g/cm^3^)	2.2–2.6	2.36
Compressive strength (MPa)	60–180	400–600
Thermal expansion coefficient (10^−5^ α_t_)	0.3–1.2	0.6–0.7
Absorptivity (%)	0.3–1.2	1.53
Porosity (%)	1.75–3.0	>5

**Table 2:** Parameters of road asphalt 50/70 [49].

Parameter	Unit	Value
Penetration at 25 °C	1/10 mm	50–70
Softening point	°C	46–54
Embrittlement temperature	°C	≤−8
Ignition temperature	°C	≥230
Solubility	% m/m	≥99.0
Mass change (absolute value)	% m/m	≤0.5
Remaining penetration at 25 °C	%	≥50
Softening point increase	°C	≤9

**Table 3:** Traffic categories.

Traffic Load	N_100_—Equivalent Standard Axle Load of 100 kN in the Entire Design Period (in Million 100 kN Axles per Lane)
TL1	0.03 < N_100_ ≤ 0.09
TL2	0.09 < N_100_ ≤ 0.50
TL3	0.50 < N_100_ ≤ 2.50
TL4	2.50 < N_100_ ≤ 7.30
TL5	7.30 < N_100_ ≤ 22.00
TL6	22.00 < N_100_ ≤ 52.00
TL7	N_100_ ≥ 52.00

**Table 4:** Compositions of the mineral mixture (MM) and mineral–asphalt mixture (MAM).

Components	WC-1		WC-2	
	% Content in			
	MM	MAM	MM	MAM
Limestone filler	9	8.5	6	5.6
0/2 quartz	16.0	15.0	22	20.7
0/4 ceramics	14.0	13.2		
0/2 dolomite	11.0	10.4	22	20.7
2/8 dolomite	12.0	11.3	30	28.3
4/8 ceramics	13.0	12.2		
8/11 dolomite	25.0	23.6	20	18.9
Bitumen 50/70		5.8		5.8
Total	100	100	100	100

**Table 5:** CA and SFE values for the mineral–asphalt mixture WC-1.

Parameter	Unit	140 °C									
**Contact Angle**	(°)	57.4	56.0	59.2	56.4	57.1	58.7	58.1	56.5	58.9	56.7
	Average (°)	57.5									
	Standard deviation (SD)	1.09									
	Coefficient of variation (CV)	0.019									
**Surface Free** **Energy**	(mJ·m^−2^)	49.41	50.25	48.33	50.01	49.59	48.63	48.99	49.95	48.51	49.83
	average (mJ·m^−2^)	49.35									
	Standard deviation (SD)	0.66									
	Coefficient of variation (CV)	0.013									
**Parameter**	**Unit**	**150 °C**									
**Contact Angle**	(°)	63.1	64.7	62.7	62.5	63.8	64.8	63	62.6	62.9	65.1
	Average (°)	63.5									
	Standard deviation (SD)	0.95									
	Coefficient of variation (CV)	0.015									
**Surface Free Energy**	(mJ·m^−2^)	45.97	44.99	46.21	46.33	45.54	44.93	46.03	46.27	46.09	44.75
	average (mJ·m^−2^)	45.71									
	Standard deviation (SD)	0.58									
	Coefficient of variation (CV)	0.013									
**Parameter**	**Unit**	**160 °C**									
**Contact Angle**	(°)	69.4	66.1	67.4	67.3	70.5	66.4	67.2	68	67.8	66.2
	Average (°)	67.6									
	Standard deviation (SD)	1.33									
	Coefficient of variation (CV)	0.019									
**Surface Free Energy**	(mJ·m^−2^)	42.1	44.13	43.33	43.39	41.42	43.95	43.46	42.96	43.09	44.07
	average (mJ·m^−2^)	43.19									
	Standard deviation (SD)	0.82									
	Coefficient of variation (CV)	0.019									

**Table 6:** CA and SFE values for the mineral–asphalt mixture WC-2.

Parameter	Unit	140 °C									
**Contact Angle**	(°)	74.7	76.1	74.5	75.5	76	74.2	74.3	74.1	76.2	75.6
	Average (°)	75.1									
	Standard deviation (SD)	0.80									
	Coefficient of variation (CV)	0.011									
**Surface Free** **Energy**	(mJ·m^−2^)	38.81	37.94	38.93	38.31	38	39.12	39.06	39.18	37.87	38.25
	average (mJ·m^−2^)	38.55									
	Standard deviation (SD)	0.50									
	Coefficient of variation (CV)	0.013									
**Parameter**	**Unit**	**150 °C**									
**Contact Angle**	(°)	79.2	80.3	79.6	80.7	79.5	80.6	78.8	80.1	80.2	78.2
	Average (°)	79.7									
	Standard deviation (SD)	0.77									
	Coefficient of variation (CV)	0.010									
**Surface Free** **Energy**	(mJ·m^−2^)	36	35.31	35.75	35.06	35.81	35.12	36.3	35.44	35.37	36.62
	average (mJ·m^−2^)	35.68									
	Standard deviation (SD)	0.49									
	Coefficient of variation (CV)	0.014									
**Parameter**	**Unit**	**160 °C**									
**Contact Angle**	(°)	82.4	83.7	83.3	84.4	82.5	83.5	84.3	82.9	82.1	82.2
	Average (°)	83.1									
	Standard deviation (SD)	0.80									
	Coefficient of variation (CV)	0.010									
**Surface Free** **Energy**	(mJ·m^−2^)	34	33.18	33.43	32.75	33.93	33.31	32.81	33.68	34.19	34.12
	average (mJ·m^−2^)	33.54									
	Standard deviation (SD)	0.50									
	Coefficient of variation (CV)	0.015									

**Table 7:** Roughness characteristics for WC-1 and WC-2.

Roughness Characteristics	WC-1			WC-2		
Temperature (°C)	140	150	160	140	150	160
Maximum peak height—Sp (µm)	132	128	102	99	88.2	81
SD	0.63	0.74	0.69	0.82	0.55	0.76
CV	0.017	0.013	0.019	0.14	0.020	0.011
Maximum valley depth—Sv (µm)	168	178	170	160	143	136
SD	0.53	0.63	0.74	0.82	0.91	0.77
CV	0.012	0.020	0.018	0.017	0.013	0.015
